# The whereabouts of presenilins: from location to mechanisms

**DOI:** 10.1002/alz70855_098886

**Published:** 2025-12-23

**Authors:** Wim Annaert

**Affiliations:** ^1^ VIB‐KU Leuven Center for Brain & Disease Research, Leuven, Belgium; KULeuven, Leuven, Belgium

## Abstract

**Background:**

The subcellular localization of γ‐secretase is to a large extent defined by the presenilin homologue: a unique sorting motif restricts PSEN2/γ‐secretase to late endosomes/lysosomes (LE/Lys) whereas PSEN1 complexes reside at the cell surface and in endosomal compartments. While these distinct locations promote substrate specificity and different amyloid β pools, they also suggest additional roles in maintaining organellar homeostasis. Using new APP knockin models with altered PSEN2 expression, we recently correlated impairments in working memory and LTP to PSEN2's role in neuronal endolysosomal homeostasis (Perdok et al., 2024). Currently we are exploring in detail how altered PSEN2 expression molecularly affects LE/Lys functions and dynamics.

**Method:**

We used nonneuronal, primary hippocampal and iPSC‐derived human neurons that are either deficient in PSEN2 or gene‐edited to express the FAD‐linked PSEN2 N141I mutation. PSEN‐deficient cells stably expressing APEX2‐tagged PSEN2 were used for proximity‐dependent biotinylation to identify the local PSEN2 interactome in LE/Lys. Hits were prioritized based on their relevance to organellar homeostasis and validated using biochemical assays and functional studies in neuronal models.

**Result:**

Proximity labeling identified novel interactors connecting PSEN2 function to LE/Lys transport regulation and nutrient sensing. Furthermore, super‐resolution microscopy was used to more precisely identify the nano‐domain organization of PSEN2 at the limiting membrane of LE/Lys. In neurons, axonal LE/Lys motility is differently affected in PSEN2KO versus FAD‐mutant PSEN2, suggesting a more complex toxic mechanism in disease context. We are currently exploiting microfluidic chambers combined with omics for a more comprehensive view on the impact of altered PSEN2 expression on the axonal compartment.

**Conclusion:**

Our data revealed new insights in how PSEN2/γ‐secretase may regulate LE/Lys homeostasis, providing novel starting points to better understand the etiology of endolysosomal dysfunctions as observed at early, preclinical stages of AD. Notably, specific FAD‐mutations also re‐locate PSEN1/γ‐secretase to LE/Lys, underscoring that knowledge gained here could aid to understand the more heterogeneous PSEN1 mutations.

